# Respiration-timing-dependent changes in activation of neural substrates during cognitive processes

**DOI:** 10.1093/texcom/tgac038

**Published:** 2022-09-13

**Authors:** Nozomu H Nakamura, Masaki Fukunaga, Tetsuya Yamamoto, Norihiro Sadato, Yoshitaka Oku

**Affiliations:** Division of Physiome, Department of Physiology, Hyogo Medical University, 1-1, Mukogawa cho, Nishinomiya, Hyogo 663-8501, Japan; Division of Cerebral Integration, Department of System Neuroscience, National Institute of Physiological Sciences, 38 Nishigonaka Myodaiji, Okazaki, Aichi 444-8585, Japan; Division of Cerebral Integration, Department of System Neuroscience, National Institute of Physiological Sciences, 38 Nishigonaka Myodaiji, Okazaki, Aichi 444-8585, Japan; Division of Cerebral Integration, Department of System Neuroscience, National Institute of Physiological Sciences, 38 Nishigonaka Myodaiji, Okazaki, Aichi 444-8585, Japan; Division of Physiome, Department of Physiology, Hyogo Medical University, 1-1, Mukogawa cho, Nishinomiya, Hyogo 663-8501, Japan

**Keywords:** exhalation, inhalation, networks, supramarginal gyrus

## Abstract

We previously showed that cognitive performance declines when the retrieval process spans an expiratory-to-inspiratory (EI) phase transition (an onset of inspiration). To identify the neural underpinning of this phenomenon, we conducted functional magnetic resonance imaging (fMRI) while participants performed a delayed matching-to-sample (DMTS) recognition memory task with a short delay. Respiration during the task was monitored using a nasal cannula. Behavioral data replicated the decline in memory performance specific to the EI transition during the retrieval process, while an extensive array of frontoparietal regions were activated during the encoding, delay, and retrieval processes of the task. Within these regions, when the retrieval process spanned the EI transition, activation was reduced in the anterior cluster of the right temporoparietal junction (TPJa, compared to cases when the retrieval process spanned the inspiratory-to-expiratory phase transition) and the left and right middle frontal gyrus, dorsomedial prefrontal cortex, and somatosensory areas (compared to cases when the retrieval process did not span any phase transition). These results in task-related activity may represent respiratory interference specifically in information manipulation rather than memory storage. Our findings demonstrate a cortical-level effect of respiratory phases on cognitive processes and highlight the importance of the timing of breathing for successful performance.

## Introduction

Breathing is a fundamental action in daily life, and alternation between phases or rhythms of breathing induces a variety of states in the body and brain. For example, the R-wave-to-R-wave interval between heartbeats shortens during inspiration and lengthens during expiration, a phenomenon known as respiratory sinus arrhythmia (Eckberg 2003; Dergacheva et al. 2010; Larsen et al. 2010). Nasal respiration entrains neural oscillations in the olfactory bulb, barrel cortex, prefrontal cortex, and hippocampus, resulting in possible consequences with respect to cognitive function (Ito et al. 2014; Zelano et al. 2016; Zhong et al. 2017; Herrero et al. 2018; Tort et al. 2018).

A recent human study demonstrated that the sampling of visual information was preferentially aligned with cortical excitability at a certain time in the respiratory phases to facilitate perceptual sensitivity (Kluger et al. 2021). Asymmetric respiratory phase locking to tactile detection was also associated with increased perceptual scores (Grund et al. 2022). The best respiratory phase locking to vision and touch occurred during the second half of the inspiratory phase and the first half of the expiratory phase, respectively. Moreover, Park et al. (2020) found that voluntary motor actions were more frequently initiated during expiration than during inspiration, and cortical readiness potential was coordinated with this behavioral observation. It is likely that the timing of respiratory phases may be involved in successful task performance.

The phases of the respiratory cycle comprise inspiration and expiration, which are derived from distinct neural mechanisms in the brainstem. The onset of inspiration, which is equivalent to an expiratory-to-inspiratory (EI) phase transition, is generated by the PreBötzinger complex (PreBötC) in the ventrolateral medulla oblongata (Smith et al. 2013; Richter and Smith 2014; del Negro et al. 2018; Oku 2022). The PreBötC is the primary inspiratory rhythm generator and is an independent, isolated complex, suggesting that the EI transition can occur in an “abrupt manner”. Meanwhile, the onset of expiration, which is equivalent to an inspiratory-to-expiratory (IE) phase transition, is widely considered to occur in a “gradual manner” because of the gate control of neural excitability (Richter and Smith 2014) and the “inspiratory off-switch” mechanism in the pons (Dutschmann and Dick 2012). These asymmetric processes might cause differential effects on functions beyond respiration.

Indeed, the timing of respiratory phases alters success rates during cognitive tasks (Zelano et al. 2016). In a cognitive task, accuracy is elevated when the presentations of test cues start with the EI transition (Perl et al. 2019), whereas accuracy is reduced when the EI transition occurs in the middle of a retrieval process (Nakamura et al. 2018). In the present study, we hypothesize that EI transition might be a key factor for the modulation of cognitive processes in the brain.

A critical question is how respiratory phases modulate brain function and subsequent cognitive performance—Does EI transition during the retrieval process change memory-dependent brain activity? Here, we collected and analyzed simultaneous functional magnetic resonance imaging (fMRI) and nasal respiration measurements from healthy participants performing a delayed matching-to-sample (DMTS) recognition memory task with a short delay as previously described (Nakamura et al. 2018). Without allowing the participants to predict the timing of test cues, we arranged tasks in two different temporal structures, Epochs A and B, which caused the test cues to be presented at variable points in the respiratory cycle. Considering that blood oxygen level-dependent (BOLD) fMRI sensitively reflects cerebral CO_2_ fluctuations and breathing patterns, our fMRI data were processed (and denoised) through multirun application of FMRIB’s independent component analysis (ICA)-based Xnoiseifier (multirun ICA-FIX) (Griffanti et al. 2017; Okamoto et al. 2020) and AFNI’s RetroTS program (Birn 2012; Murphy et al. 2013).

## Materials and methods

### Subjects

The participants in this experiment were 31 healthy volunteers. None of the subject was regularly taking medication, and none had a known history of respiratory, cardiovascular, endocrine, neurological, or psychiatric disease. Written informed consent was obtained from all participants. All participants were scored as right-handed according to the Edinburgh Handedness Inventory (Oldfield 1971). Six subjects were excluded (two made button-press responses in under 95% of the task and four answered correctly less than 80% of the time). In total, 25 healthy subjects (11 males and 14 females; age: 22.0 ± 0.5 years, range: 20–31 years) were included for further analyses. All procedures performed on humans were in accordance with the Declaration of Helsinki (Ethical Principles for Medical Research Involving Human Subjects) and the Ethical Guidelines for Medical and Health Research Involving Human Subjects, Japan, and all procedures were approved by the Ethics Committee of Hyogo College of Medicine, Japan (No. 1825) and the Ethics Committee of the National Institute for Physiological Sciences, Japan (18A001, 19A001, 20A004, 21A004).

### Respiratory apparatus

Inspiration and expiration during the respiratory cycle were continuously recorded via a flow sensor nasal cannula (Flow Nasal Cannula A, 1 m, Atom Medical, Japan) extended with a polyvinyl chloride tube (6 m length, id: 1 mm, od: 3 mm) and equipped with a differential pressure transmitter (Model KL17, Nagano Keiki, Japan). The respiratory waveforms and signals used as the present visual information for the task were sampled at 1 kHz using the PowerLab data acquisition system (PowerLab, AD Instruments, Dunedin, New Zealand) and were processed online using LabChart software (LabChart 7.1, AD Instruments). Before each experiment, the air pressure in the MRI room was measured to establish a baseline level. Of note, we adjusted the respiratory waveforms to compensate for a constant time-lag (0.27 s) because the respiratory waveforms in this experiment were continuously delayed by the nasal cannula and a 6-m tube extended in the experiment.

### Behavioral task paradigm

**Fig. 1:**
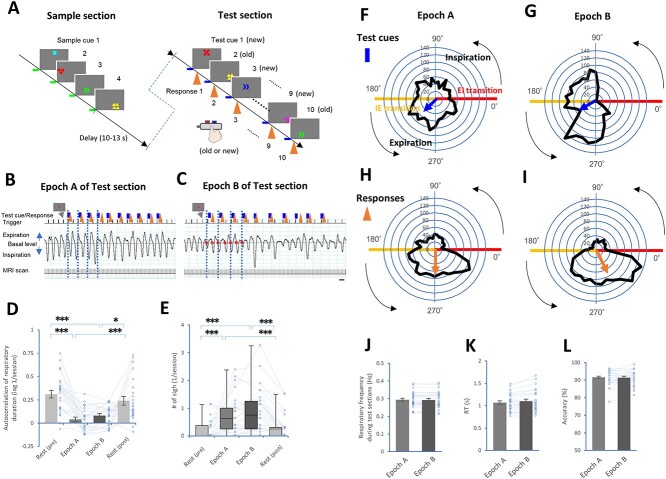
The DMTS task and motor responses were modulated along with the respiratory cycle. (A) A task paradigm consisting of four sample cues (green lines), a delay, and ten test cues (blue lines). Button presses (orange arrows) were based on the observed decisions (see Supplementary Fig. 1). (B and C) Plots showing the respiratory waveforms (middle), along with the timing of the test cues, responses, triggers (top), and MRI scans (0.8 s each, bottom) in Epochs A (B) and B (C). (D and E) Plots showing the autocorrelation (one lag) of the respiratory duration (D) and the number of sighs (E) during Epochs A and B, and the resting periods. (F-I) Black-outlined polygons form histograms of the test cues (F and G) and button-press responses (H and I) in Epochs A and B according to their positions in degrees along phases of the respiratory cycle: angles form 0° to 180° represent inspiration, and angles from 180° to 360° represent expiration. The EI transitions are marked with red lines, and the IE transitions are marked with yellow lines. The directions of the arrows in the circles (test cues: blue, responses: orange) indicate the mean of the relative phase along the circular respiratory phases, and a shorter length indicates greater dispersion of the relative phase (see Supplementary Fig. 2, Supplementary Table 1). (J-L) Plots showing respiratory frequency (J), RT (K), and accuracy (L) between Epochs A and B. A scale bar in C indicates 3 s (B and C). ^*^P < 0.05 and ^***^P < 0.005 (post hoc pairwise comparisons).

A DMTS version of a visual recognition memory task with a short delay was employed as previously described in the study by Nakamura et al. (2018) with minor modifications. The DMTS task consisted of a sample block, a delay block, and a test block structured according to a standard DMTS protocol (Fig. 1A) (Eichenbaum et al. 2007; Nakamura and Sauvage 2016). Task paradigms were created in NBS Presentation® software (Presentation 18.3, Neurobehavioral Systems). The DMTS task required the memorization and recognition of visual cues comprising a symbol (configuration), its color, its position, and the number instances of the symbols on a projector screen (a liquid crystal display projector, CP-SX12000J, Hitachi, Japan) in the MRI scanning room. The symbol (configuration) was a circle, triangle, rectangle, cross, crescent, or heart; its color was red, blue, green, yellow, pink, or sky-blue; the number of symbols was one, two, three, four, five, or six; and the symbol(s) were positioned at the center, right, left, top center, bottom right, or bottom left of the screen. Thus, there were 1,296 (6 × 6 × 6 × 6 variables) possible combinations (Supplementary Fig. 1). Subjects were instructed to fix their eyes on the white cross at the center of the screen. At the start of the sample section, the white cross turned green, and each subject was then exposed to a series of four visual cues (i.e. sample cues) displayed one at a time on the screen.

After a delay (10–13 s), the white cross turned red, and then each subject was tested ten separate times for the ability to distinguish between visual cues presented during the sample section (“old” or match cues) and “new” (or nonmatch) cues selected from the 1,292 out of 1,296 stimuli that were not presented during the sample section (Fig. 1A). After each test cue, the subjects were required to indicate whether the presented cue was the same as one of four sample cues presented and then to press the corresponding button. An “old” (or match) cue was defined as a cue that matched one of the sample cues in all four characteristics: symbol (configuration), color, position, and number of figures. A “new” (or nonmatch) cue was defined as a cue that did not perfectly match any of the sample cues in this manner. The subjects pressed one of two buttons using their left thumb once they had identified the cue shown during the test section as an “old” or “new” cue. Five “old” and five “new” cues were presented in random manner during the test section. Before the experiments, the subjects were instructed on how to perform the task and told to breathe in a relaxed, natural manner during the task.

Each participant memorized 4 sample cues and recognized 10 test cues per epoch; there were 16 epochs in total. We recognized that it may be necessary to prevent participants from predicting the timing of test cues. Accordingly, we designed two epochs, i.e. Epochs A and B to create variability in the interstimulus interval (ISI) between test cues (see Nakamura et al. 2018).

In Epoch A, the ISI was 6 s, with one of three different time lags in random order (0, 350, or 700 ms after a trigger signal, Fig. 1B). In Epoch B, test cues were set to occur at specific times during the participant’s respiratory cycles, i.e. an EI transition or an IE transition, with one of three different time lags in random order (0, 350, or 700 ms after a trigger signal, Fig. 1C). For the detection of the EI or IE transition point during Epoch B, we used a real-time monitoring method with a filtered respiratory waveform. The sampled waveforms were processed with a low-pass (< 2 Hz) filter to eliminate the effect of noise and isolate the respiratory cycle. A trigger signal was immediately driven by the timing of the EI or IE transition when the filtered waveform crossed the basal level (i.e. each trigger signal was set once every two EI transitions or once every two IE transitions). Moreover, each trigger signal had an additional time lag of approximately 0.67 s (0.27 s caused by the 6-m length of the tube and 0.4 s arising from mechanical causes) from the EI or IE transition point in the raw waveforms. Then, the eight instances of Epoch B consisted of four EI-transition-dependent and four IE-transition-dependent epochs in a pseudorandom manner.

Each subject performed 16 epochs (eight of Epoch A and eight of Epoch B), in which a total of 160 test cues was discriminated as either old or new. Prior to the experiments, the subjects were not informed of the timing of the test cues during the tasks. We confirmed that subjects did not control their breathing intentionally during the task, since no subjects reported awareness of the cues being locked to their own respiratory cycle, or of intentionally adjusting their breathing to correspond with the timing of cue exposure to improve their performance.

### Behavioral data analysis

During the experiments, we measured the following parameters: (i) cognitive parameters including the time of cue exposure, time of button pressing, and accuracy, as captured by NBS Presentation software; (ii) respiratory parameters including the onset of every inspiration and expiration in the raw respiratory waveform, as captured by LabChart software (Fig. 1B and C); and (iii) BOLD fMRI data. At a preset level, the onsets of every inspiration and expiration were defined as the time at which the flow first crossed the baseline level and deviated from it by over ±2 SD to ensure that the level exceeded the baseline noise level. Thus, the onsets of inspiration and expiration corresponded to the EI transition and IE transition, respectively. The series of respiratory parameters was synchronized with the series of cognitive parameters, and then synchronized with each run of fMRI scans.

Regarding respiratory parameters, the autocorrelation of respiratory durations at one lag and the number of sighs were calculated during resting periods before and after the tasks (Rest (pre) and Rest (post)), Epoch A, and Epoch B (Fig. 1D and E). Sighs were defined by breaths with a nasal tidal pressure amplitude at least twice as large as the mean tidal pressure amplitude during each period (Vlemincx et al. 2012, 2013).

Within histograms of test cues and button-press responses between Epochs A and B along the circular respiratory phases (Fig. 1F-I), the arrows ($\overrightarrow{M}$) are averaged over all test cues or responses along with circular phases in each histogram:

$$ \overrightarrow{M}=\frac{\sum_{i=1}^N\overrightarrow{Pi}}{\mathrm{N}} $$

where $\overrightarrow{Pi}$is a unit-length vector in the direction of the i^th^ estimation of the relative phase. The direction of the arrow indicates the mean of the relative phase, while a shorter length indicates greater dispersion of the relative phase (Kennerley et al. 2002). Then, the following parameters were calculated along the circular respiratory phases between Epochs A and B: phase shifts and changes in standard deviation from the test cues to responses across individual subjects (Supplementary Fig. 2).

**Fig. 2:**
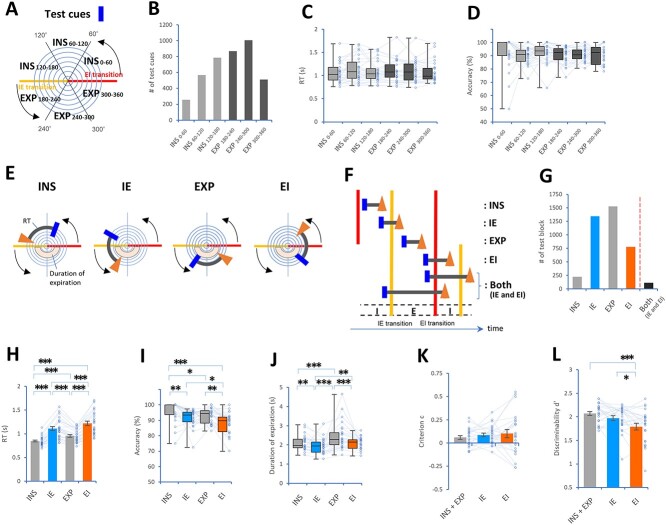
Respiration modulated the retrieval process in the DMTS task. (A) Drawing showing six conditions of test cue exposures along phases of the respiratory cycle: (i) test cues started from 0° to 60° (INS 0–60), (ii) test cues started from 60° to 120° (INS 60–120), (iii) test cues started from 120° to 180° (INS 120–180), (iv) test cues started from 180° to 240° (EXP 180–240), (v) test cues started from 240° to 300° (EXP 240–300), and (vi) test cues started from 300° to 360° (EXP 300–360). (B-D) Plots showing the number of test cues (B), RT (C), and accuracy (D) among the six conditions. (E and F) Drawing showing conditions of combinations between test blocks and phases of the respiratory cycle: (i) test blocks within the inspiratory phase (INS condition), (ii) test blocks encompassing the IE transition point (IE condition), (iii) test blocks within the expiratory phase (EXP condition, (iv) test blocks encompassing the EI transition point (EI condition), and (v) test blocks encompassing both IE and EI transitions (a condition designated “Both”). (G-J) Plots showing the number of test blocks (G), RT (H), accuracy (I), and duration of expiration (J) in the four conditions. (K and L) Plots showing the criterion c (K) and discriminability d′ of familiarity-based memory (I) among the conditions. ^*^P < 0.05 and ^*^^*^^*^P < 0.005 (post hoc pairwise comparisons).

A “test block” was defined as the period from a test cue presentation to a motor response. The RT was calculated as the time scale between a test cue exposure and a response. We divided the timing of test cues into four conditions throughout the respiratory cycle (Fig. 2A and B): (i) test cues distributed in the first part of the inspiratory phase (INS 0–60); (ii) test cues in the second part of the inspiratory phase (INS 60–120); (iii) test cues in the last part of inspiration (INS 120–180); (iv) test cues in the first part of the expiratory phase (EXP 180–240); (v) test cues in the second part of the expiratory phase (EXP 240–300); and (vi) test cues in the last part of the expiratory phase (EXP 300–360). The test blocks were also divided into conditions (Fig. 2E-G): (i) test blocks fitting within inspiration (INS condition); (ii) test blocks encompassing the IE transition (IE condition); (iii) test blocks fitting within expiration (EXP condition); and (iv) test blocks encompassing the EI transition (EI condition). Importantly, test blocks encompassing both IE and EI transitions (a condition designated “Both”) were excluded from further analysis because the number of individual test blocks was too small (Fig. 2G).

Regarding familiarity-based recognition, we calculated discriminability d′, which is the separation factor between the “new” and “old” distributions in signal detection theory (Stanislaw and Todorov 1999; Yonelinas 2002; Arshamian et al. 2018). In brief, this factor is estimated from *z* scores using the following equation: d′ = z (hit) + z (correct rejection) = z (hit)—z (false alarm) (Stanislaw and Todorov 1999). Meanwhile, the decision criterion c was defined as the negative value of half of the sum of z (hit) and z (false alarm). Notably, criterion c is considered the degree of bias strength for the discrimination between “old” and “new” figures.

### Behavioral statistical analysis

Across individual subjects, we tested for normality and sphericity using the Shapiro–Wilk normality test and the Mauchly test for sphericity. We used a two-tailed paired *t* test, one-way or two-way repeated-measures ANOVA, post hoc pairwise comparisons using a paired *t* test with the Bonferroni correction, a two-tailed one-sample *t* test against zero, Pearson’s product–moment correlation analysis, and repeated-measures correlation analysis (rmcorr). If the assumptions of normality and sphericity were violated, we performed the nonparametric Friedman test followed by post hoc pairwise comparisons using the Wilcoxon signed-rank test with the Bonferroni correction and applied the Greenhouse–Geisser correction for departure from sphericity. All statistical analyses were performed using R version 3.6.1 software (R Core Team, R Foundation for Statistical Computing, Vienna, Austria, 2019, https://www.R-project.org/).

### MRI

fMRI data were acquired using a 3.0 T MRI scanning machine (Magnetom Verio, Siemens Healthineers AG, Erlangen, Germany) with a 32-element phased array head coil. fMRI was performed using a multiband gradient echo-type of eco planar imaging (GE-EPI) sequence (Moeller et al. 2010) with a modified version of the Human Connectome Project (HCP) protocol (Glasser et al. 2013) (TR/TE = 800/31 ms; FA = 55°; FOV = 208 x 208 mm; matrix size = 104 x 104; 72 transverse slices with thickness = 2 mm to cover the whole brain; echo spacing = 0.75 ms; multiband acceleration factor = 8; and a posterior-to-anterior phase-encoding direction). Two spin-echo EPI datasets with reversed phase-encoding directions were also acquired (three volumes with each phase-encoding direction) with the same geometric and echo spacing parameters (TR = 7,700 ms; TE = 60 ms; FA = 78°; and refocus FA = 160°) as the GE-EPI sequence. These datasets were used to correct susceptibility-induced distortion with FSL’s topup tool (Andersson et al. 2003). Whole-brain high-resolution T1-weighted anatomical magnetization-prepared rapid-acquisition gradient echo (MP-RAGE) MRI was also performed at 0.9-mm isotropic resolution for each subject (TR/TE/TI = 2,000/2.18/962 ms; FA = 9°; FOV = 230 × 230 mm; matrix size = 256 × 256; and 208 sagittal slices in a single slab with thickness = 0.8 mm). Each subject performed DMTS tasks consisting of two of Epoch A and two of Epoch B in randomized order during one run of fMRI scans (825 scans, 11 min). In total, each subject performed 16 epochs of the task (four runs) in the MRI machine. To maintain alertness during the remaining fMRI scans following four epochs of the task, each participant continued to perform an attentional task, in which either button was pressed when the white cross at the center of the screen turned blue. The projector screen was located outside and behind the scanner and projected stimuli through a waveguide to a translucent screen that the participants viewed via a mirror attached to the head coil of the MRI scanner.

### fMRI data processing

The fMRI images were preprocessed using the HCP minimum preprocessing pipeline 4.2.0 with minor modifications (Glasser et al. 2013). The following processes were performed on volume data: corrections for image distortions induced by gradient magnetic field nonlinearity and static magnetic field susceptibility; nonlinear image normalization to the Montreal Neurological Institute (MNI) space; intensity normalization; and independent component analysis (ICA)-based denoising of time series data concatenated from four runs using multirun FMRIB’s ICA-based Xnoiseifier (multirun ICA-FIX) (Griffanti et al. 2017; Okamoto et al. 2020).

Regarding the procedure of multirun ICA-FIX, spatially independent components were first extracted from a time-series dataset formed by concatenating four demeaned, variance-normalized single-run datasets. They were then manually classified into “signal” and “noise” components according to the hand classification criteria (Griffanti et al. 2014, 2017). In the cleanup step, motion confounds were not regressed out, because of concern about “phantom motion” induced by respiration (Glasser et al. 2018). Finally, the cleaned data were split back into single-run data to return the time series to its original state without noise. These processes were carried out using FSL 6.0.4 (https://fsl.fmrib.ox.ac.uk/fsl/fslwiki); Connectome Workbench 1.4.2 (https://www.humanconnectome.org/software/connectome-workbench); HCP-gradunwarp 1.2.0 (https://github.com/Washington-University/gradunwarp); FIX 1.06.15 (https://git.fmrib.ox.ac.uk/fsl/fix/-/tree/1.06.15); R 3.6.3 (https://www.r-project.org/); and MATLAB Runtime R2016a (MathWorks, Natick, MA, USA) running on CentOS Linux 7.9 (https://www.centos.org/).

The cleaned fMRI images were analyzed using the Statistical Parametric Mapping software (SPM12, UCL Queen Square Institute of Neurology, London, UK, https://www.fil.ion.ucl.ac.uk/spm/) implemented in MATLAB R2018b. The first ten volumes of each fMRI scan were discarded to allow the MR signal to reach a state of equilibrium. All processed fMRI data were spatially smoothed in 3D using a Gaussian kernel with a 6-mm full width at half maximum (FWHM) to reduce the remaining noise and respiratory effects.

Variations in RVT causes fluctuations in arterial CO_2_ concentrations that influence BOLD fMRI signals (Birn 2012; Murphy et al. 2013). To take into account such global respiratory artifacts in BOLD fMRI signals, each subject’s respiratory waveforms as measured by the nasal cannula were entered in AFNI’s RetroTS program as the regressor RVT (Birn et al. 2006, 2008; Power et al. 2020). In our preliminary study, we found a positive correlation between the RVT parameters calculated from respiratory waveforms of the nasal cannula and the pneumatic belt around the chest (Supplementary Fig. 3). This parameter was averaged within each time window (0.8 s) with a 5.6-s delay before usage in fMRI data analysis carried out across windows (Birn et al. 2009).

### fMRI data analysis

**Fig. 3:**
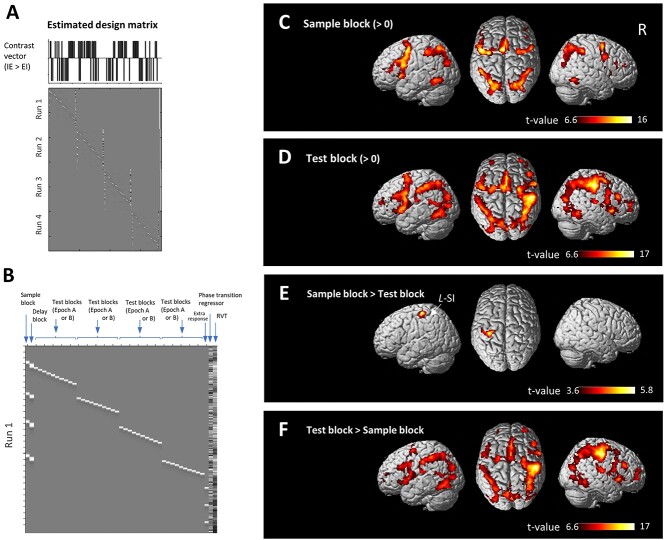
fMRI activity during the sample and test blocks. (A) Matrix showing a representative estimated design matrix (four fMRI runs, bottom panel) with the contrast vector between the IE condition and the EI condition (i.e. IE > EI, top panel) at an individual level according to single participant performance. (B) Matrix showing a representative paradigm containing the sample blocks, delay blocks, 40 test blocks, extra button-press response, phase transition regressor, and RVT regressor during a single fMRI run. (C-F) Images showing brain regions that exhibited fMRI activity during the sample block (C), test block (D), and fMRI activity contrasting the sample block in contrast to the test block (sample block > test block, E), and the test block in contrast to the sample block (test block > sample block, F). fMRI activity is projected onto the cortical surface at an SPM{t} threshold of *P* < 0.05 with family-wise error (FWE) correction at the peak level (C,D,F) and cluster level (E) for the whole brain.

A general linear model was fitted to the fMRI data for each participant (Friston et al. 1994; Worsley and Friston 1995). The time series of the BOLD signal was modeled with boxcar functions corresponding to task components, convolved with the canonical hemodynamic response function. Four runs of fMRI scans were conducted in each participant performing the task (i.e. 16 epochs containing a total of 64 sample and 160 test cues). During each run of fMRI scans containing four epochs, a matrix of task components was individually designed as follows: a sample block (the period from the first sample cue to fourth sample cue), a delay block (the period from the fourth sample cue to the presentation of the red cross), 40 test blocks, and an extra response block (Fig. 3A). The appearance of the IE and EI transition points was converted into a time series with 0.8 s temporal resolution, and then both series were integrated as a single regressor (the phase transition regressor, i.e. [IE transition, EI transition] = [1, −1]). The time windows for each voxel were processed with a high-pass filter with a threshold of 1/128 Hz. As the traditional AR(1) plus noise model can fail to whiten data with a short TR, temporal autocorrelations were modeled and estimated from the pooled active voxels by the FAST model and were used to whiten the data (Corbin et al. 2018). This alternative pre-whitening method is reported to perform better than SPM’s default (Olszowy et al. 2019). The contrast estimates for each condition against the baseline were evaluated using linear contrasts (Kitada et al. 2019).

Contrast images of the individual-level analysis, which represented the normalized task-related increment of the MR signal of each participant, were used for the group-level analysis. In particular, the three conditions were estimated by contrasts according to individual participants’ performance (see Fig. 3B). The resulting set of voxel values for each contrast constituted the SPM {t}. The statistical threshold for the peak test (peak level) and for the spatial extent test on the clusters (cluster level) was set at *P* < 0.05 corrected for family-wise error (FWE) at the whole brain level.

## Results

### Respiration was modulated during the task

Twenty-five healthy participants performed the DMTS task, which required the memorization and recognition of visual cues containing a symbol (configuration), its color, its position, and the number of instances of the symbols (Fig. 1A, Supplementary Fig. 1). Then, to prevent participants from predicting the timing of test cues, we designed Epochs A and B, which varied the timing of the test cues (Fig. 1B and C, see Methods). As calculated by the Shapiro–Wilk normality test, respiratory correlated variability (i.e. autocorrelation of respiratory duration) had a normal distribution in Epochs A and B and resting periods before and after the tasks (i.e. Rest (pre) and Rest (post)), whereas sigh frequency did not. We found significant differences in the autocorrelation of respiratory duration (*n* = 25 subjects, *P* < 0.00001, Mauchly tests for sphericity; *F*(3, 72) = 14.73, *P* < 0.00001 with Greenhouse–Geisser correction for departure from sphericity) and sigh frequency (*χ*^2^(3) = 35.54, *P* < 0.00001, Friedman test). Post hoc pairwise comparisons showed that Epoch A and Epoch B exhibited lower autocorrelation of respiratory duration (*n* = 25 subjects, paired *t* test with Bonferroni correction, Fig. 1D) and higher sigh frequencies (Wilcoxon signed-rank test with Bonferroni correction, Fig. 1E) than the resting periods. These results revealed that respiration was spontaneously modulated during the DMTS task.

### Button-press responses were modulated along with respiratory cycles

We found distinct pattern histograms for test cue exposure (*n* = 1,985, Fig. 1F; *n* = 2,001, Fig. 1G) and button-press response (*n* = 1,985, Fig. 1H; *n* = 2,001, Fig. 1I) along the respiratory cycle between Epochs A and B. The data were plotted in degrees, where a full circle represents one complete respiratory cycle. Of note, the mean durations of inspiration and expiration were different (inspiration (0–180°): 1.46 ± 0.05 s; expiration (180–360°): 2.09 ± 0.08 s). The test cues in Epoch A were relatively evenly scattered throughout the respiratory cycle (Fig. 1F), while the distribution of the test cues in Epoch B was skewed between inspiration and expiration because of the exposure to the test cue settings (Fig. 1G). However, the histograms of the motor responses had similar shapes between Epochs A and B (Fig. 1H and I). A finer-gained analysis showed that the timing of motor responses was aligned preferentially with respiratory phases, even though the sampling of visual cues differed with respect to the respiratory phases (Supplementary Fig. 2, Supplementary Table 1). Meanwhile, there were no differences in respiratory frequency (*n* = 25 subjects; *t*(24) = 1.36, *P* = 0.2, paired *t*-test, Fig. 1J), RT (*t*(24) = 1.96, *P* = 0.06, Fig. 1K), or accuracy (*t*(24) = 0.26, *P* = 0.8, Fig. 1L) between Epochs A and B.

### The retrieval process was modulated by respiration

To determine whether the detection of test cues showed effects that depended on the respiratory phase, we used Epochs A and B together and divided the timing of the test cues into six conditions throughout the respiratory cycle (Fig. 2A and B): (i) INS 0–60; (ii) INS 60–120; (iii) INS 120–180; (iv) EXP 180–240; (v) EXP 240–300; and (vi) EXP 300–360 (see section Methods). Then, the RT and accuracy of the individual test blocks were averaged within each subject for each condition. The Shapiro–Wilk test did not show normality in the RT and accuracy. There was no difference in RT (*n* = 25 subjects; *χ*^2^(5) = 4.95, n.s., Friedman test, Fig. 2C), or in accuracy (*χ*^2^(5) = 7.65, n.s., Fig. 2D). The six subdivisions of test cues along the respiratory cycles were not associated with any performance differences.

A “test block” was defined by the period from a test cue presentation to a motor response, and then ten test blocks were classified into the following conditions (Fig. 2E-G): (i) the INS condition; (ii) the IE condition; (iii) the EXP condition; and (iv) the EI condition (see section Methods). Then, the RT and accuracy of the individual test block were averaged per subject for each condition. The duration of expiration during the test block was also calculated per subject. The RT was normally distributed in all four conditions, whereas accuracy and the duration of expiration were not normally distributed. We found significant differences in the RT (*n* = 25 subjects, *P* = 0.00001, Mauchly tests for sphericity; *F*(3, 72) = 81.54, *P* < 0.00001 with Greenhouse–Geisser correction for departure from sphericity), accuracy (*χ*^2^(3) = 21.52, *P* = 0.00008, Friedman test), and expiration duration (*χ*^2^(3) = 36.60, *P* < 0.00001, Friedman rank sum test). Post hoc pairwise comparisons showed that the EI condition (i.e. the test block encompassing an EI transition point) exhibited the highest RT, the IE condition (i.e. the test block encompassing an IE transition point) the second-highest RT, and the EXP condition the third-highest RT (paired *t* test with Bonferroni correction, Fig. 2H). Moreover, accuracy was lowest in the EI condition, and lower accuracy was observed in the IE condition than in the INS condition (Wilcoxon signed-rank test with Bonferroni correction, Fig. 2I). Expiration duration was longest in the EXP condition, and a longer expiration duration was observed in the EI condition than in the IE condition (Wilcoxon signed-rank test with Bonferroni correction, Fig. 2J). These results replicated our previous findings that the EI condition extended RT and decreased accuracy (Nakamura et al. 2018).

### Familiarity-based recognition was modulated by respiration

To further determine whether respiration modulates other memory components, we estimated the criterion c and discriminability d′ as familiarity parameters for recognition memory (Stanislaw and Todorov 1999; Yonelinas 2002; Arshamian et al. 2018). Since the effects of the IE and EI conditions (i.e. the test blocks encompassing phase transition points) were prioritized in the present study, the INS condition was combined with the EXP condition for further analysis. The Shapiro–Wilk test showed normality in criterion c and discriminability d′. No main effect was observed in criterion c among the INS + EXP, IE, and EI conditions (*n* = 25 subjects, *P* = 0.01, Mauchly tests for sphericity; *F*(2, 48) = 0.72, n.s., with Greenhouse–Geisser correction, Fig. 2K), whereas we found a significant main effect in the discriminability d′ (*F*(2, 48) = 9.87, *P* = 0.0003, two-way repeated-measures ANOVA, Fig. 2L). Post hoc pairwise comparisons using a paired *t* test with the Bonferroni correction showed that the EI condition exhibited the lowest discriminability d′. Our results showed that the EI condition reduced familiarity-based recognition.

### The EI condition was associated with reduced activations in the right TPJa, right MFG, and dACC/SMA

Considering the respiratory effects contained in BOLD fMRI, our fMRI data were carefully denoised via multirun ICA-FIX (Griffanti et al. 2017; Okamoto et al. 2020), and AFNI’s RetroTS program was applied to regress out RVT (Birn 2012; Murphy et al. 2013) (Fig. 3A and B, Supplementary Fig. 3). Furthermore, a regressor of the variability between phase transition points was added for the analyses during all fMRI runs to exclude any remaining respiratory effect (Fig. 3B). The sample block (the period from the first sample cue to the fourth sample cue) was associated with fMRI activity in extensive frontoparietal regions, the inferior frontal gyrus (IFG), the middle frontal gyrus (MFG), the frontal operculum, the anterior insula, the dorsal part of the anterior cingulate cortex (dACC), the presupplementary motor area (PreSMA), the temporal gyrus, the hippocampus, the supramarginal gyrus (SMG), the superior parietal lobule, caudate, and the thalamus (Fig. 3C, Supplementary Table 2); this set of regions is similar to the set activated by the delay block (Supplementary Fig. 4, Supplementary Table 3). The test block was associated with fMRI activity in the IFG, MFG, MI, frontal operculum, dACC, PreSMA, midcingulate cortex, posterior cingulate cortex, temporal gyrus, fusiform gyrus, postcentral gyrus, occipital fusiform gyrus, middle occipital gyrus, and caudate (Fig. 3D, Supplementary Table 4). Contrasting the sample block with test block in the order, Sample block > Test block showed fMRI activity in the left primary somatosensory cortex (SI, Fig. 3E, Supplementary Table 5), whereas the opposite contrast (Test block > Sample block) showed fMRI activity in a set of frontoparietal regions similar to the set activated by the test block (Fig. 3F, Supplementary Table 6).

**Fig. 4:**
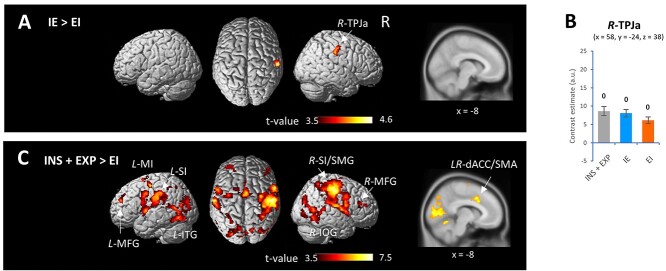
Regions showing EI-transition-specific fMRI activity during the test block. (A) Images showing brain regions that exhibited fMRI activity in the IE condition in contrast to the EI condition (IE > EI). (B) Plots showing contrast estimates among the INS + EXP, IE, and EI conditions in the right TPJa (MNI coordinates: *x* = 58, *y* = −24, *z* = 38). (C) Images showing brain regions that exhibited fMRI activity in the INS + EXP condition in contrast to the EI condition (INS + EXP > EI). fMRI activity was exhibited in the region of interest at an SPM{t} threshold of *P* < 0.05 with cluster-level FWE correction for the whole brain. ^0^*P* < 0.00001 (one-sample *t*-test against zero), a.u.: arbitrary unit.

We also addressed the question of whether the EI-transition-dependent effect was functionally and anatomically represented by neural substrates. In line with the familiarity-based recognition results (Fig. 2L), contrasting the IE condition with the EI condition in the order, IE > EI revealed fMRI activity in the right SMG (Montreal Neurological Institute (MNI) coordinates: *x* = 58, *y* = −24, *z* = 38, Fig. 4A, Supplementary Table 7), which was equivalent to the anterior part of the temporoparietal junction (TPJa) (Mars et al. 2012; Igelström et al. 2015). However, the opposite contrast (EI > IE) did not reveal any fMRI activity. In the right TPJa, the three conditions (INS + EXP, IE, and EI) had significant estimated contrasts with the baseline values (contrast estimates relative to baseline; INS + EXP: *t*(24) = 6.93, *P* < 0.00001, IE: *t*(24) = 6.70, *P* < 0.00001, EI: *t*(24) = 6.70, *P* < 0.00001, one-sample *t* test against zero, Fig. 4B). Contrasting the INS + EXP condition with the EI condition (INS + EXP > EI) revealed fMRI activity in the left/right MFG, left postcentral gyrus (SI)/central operculum, left/right dACC/supplementary motor area (SMA), left MI, right SI/SMG, left/right inferior temporal gyrus, and right inferior occipital gyrus (Fig. 4C, Supplementary Table 7). The reverse contrasts, EI > INS + EXP did not reveal any fMRI activity. These results revealed that the test block encompassing the EI transition (i.e. EI condition) had reduced activation of the right TPJa, the left and right MFG, and the dACC/SMA.

### Activation in the right TPJa, right MFG, and dACC/SMA was modulated by respiration

**Fig. 5:**
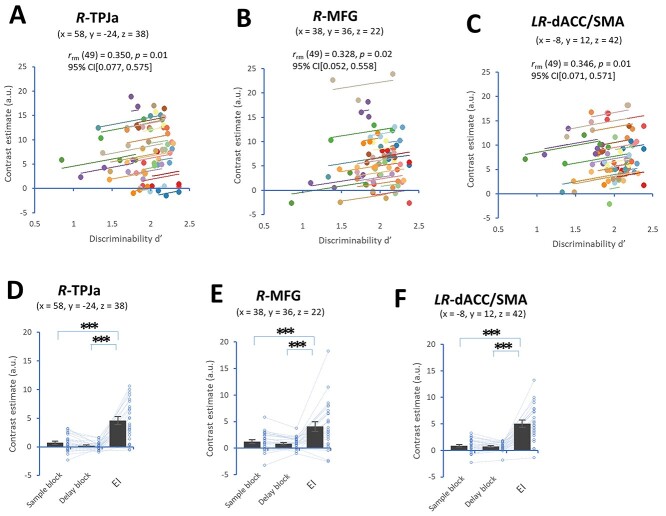
Region-of-interest analysis of fMRI activity. (A-C) Plots showing positive correlations at the within-individual level (rmcorr) between the discriminability d′ of familiarity-based memory and contrast estimates in the right TPJa (A), right MFG (B), and dACC/SMA (C). (D-F) Plots showing contrast estimates in the right TPJa (D), right MFG (E), and dACC/SMA (E) among the sample block, delay block, and EI condition (part of the test block). ^*xs**^P < 0.005 (post hoc pairwise comparisons), a.u.: arbitrary unit.

Using the rmcorr package and contrast estimates in the TPJa, MFG, and dACC/SMA during the test block, we found significant positive correlations at the within-individual level between the discriminability d′ and contrast estimates among the INS + EXP, IE, and EI conditions in the right TPJa (*n* = 25 subjects; *r*_rm_ (49) = 0.350, *P* = 0.01, 95% CI[0.077, 0.575], Fig. 5A), the right MFG (*r*_rm_ (49) = 0.328, *P* = 0.02, 95% CI[0.051, 0.558], Fig. 5B, and dACC/SMA (*r*_rm_ (49) = 0.346, *P* = 0.01, 95% CI[0.071, 0.571], repeated-measures correlation, Fig. 5C). However, there was no correlation between the discriminability d′ and contrast estimates in the left MFG (*r*_rm_ (49) = 0.199, n.s., 95% CI[−0.094, 0.460]). In comparisons with the sample and delay blocks and the EI condition (part of the test block), significant main effects were observed on contrast estimates in the right TPJa (*n* = 25 subjects, *P* < 0.00001, Mauchly tests for sphericity; *F*(2, 48) = 38.67, *P* < 0.00001 with Greenhouse–Geisser correction), right MFG (*P* < 0.00001, Mauchly tests for sphericity; *F*(2, 48) = 17.49, *P* = 0.0002 with Greenhouse–Geisser correction), and dACC/SMA (*P* < 0.00001, Mauchly tests for sphericity; *F*(2, 48) = 45.37, *P* < 0.00001 with Greenhouse–Geisser correction). Post hoc pairwise comparisons showed that the EI condition in the test block exhibited higher contrast estimates in the right TPJa (Fig. 5D), right MFG (Fig. 5E), and dACC/SMA (Fig. 5F) than the sample and delay blocks. These results showed that the test block containing an EI transition point (i.e. the EI condition) was associated with activation in the right TPJa, right MFG, and dACC/SMA, while these specific regions were rarely activated during the sample block or delay block.

## Discussion

These findings brought evidence that respiration can modulate the activation of specific brain regions during memory processes. In line with our previous study using the DMTS task (Nakamura et al. 2018), task performance fluctuated with the degrees of respiratory phases and reduced when retrieval and recognition processes encompassed EI transition (EI condition). Moreover, button-press responses were aligned preferentially with specific times in the respiratory cycle. Notably, changes specific to the EI transition were represented by activation of the right TPJa, right MFG, and dorsomedial prefrontal cortex (i.e. dACC/SMA), which were less activated during the retrieval process. However, the EI-transition-dependent activation was higher than that during the encoding process or the delay period of the task. Meanwhile, familiarity-based recognition was in response to activation in the right TPJa, right MFG, and dorsomedial prefrontal cortex at the within-individual level: hence, the retrieval process excluding an EI transition point (i.e. INS + EXP and IE conditions) might show increased familiarity-based recognition owing to increased activation of the networks. These results suggest that the coordination between the timing of respiration and specific cortical networks could be a key driver in modulating brain function, thereby influencing subsequent task performance.

In the present study, task performance was unchanged by the detection of visual information during the respiratory phases. Human studies have demonstrated that visual and tactile detection can be aligned with specific times in the respiratory cycle (Kluger et al. 2021; Grund et al. 2022), whereas animal studies have shown that perceptual detection, such as sniffing, whisking, and odor discrimination, induces respiratory phase locking to neural activity in the olfactory bulb and somatosensory cortex (Buonviso et al. 2006; Cury and Uchida 2010; Shusterman et al. 2011; Deschênes et al. 2016). However, performing cognitive tasks not only recruits sensory detection but also demands many different categories of processes, e.g. accessing memory storage and making decisions, of which memory retrieval processes may require especially stable and organized brain states, as with the case of voluntary actions during cognitive tasks; this is distinct from the case of simple, externally triggered actions (Park et al. 2020). We propose that the respiratory modulation of perceptual sampling might be overridden by respiratory alignments during recognition and retrieval processes.

The DMTS task or working memory task elicited increased activation in the hippocampus and parahippocampal region during encoding, whereas retrieval corresponded to increased activation in the dACC and dorsolateral prefrontal cortex in numerous previous fMRI studies (Monk et al. 2002; Zhang et al. 2008; Daniel et al. 2016; Schon et al. 2016), in which task paradigms and fMRI contrasts were different from those in the current experiment. Our fMRI data showed that the encoding process activated part of the hippocampus, the IFG, and the temporal gyrus, while the retrieval process after a brief delay activated the IFG, MFG, dACC, and fusiform gyrus, but not the hippocampus, suggesting that the retrieval process of our task paradigm was biased toward the hippocampus-independent process of familiarity rather than recollection (Eichenbaum et al. 2007; Yonelinas et al. 2010; Koen et al. 2017). Familiarity, which reflects a more global measure of memory strength and recency judgment, is similar to conceptual implicit memory in that these processes have common mechanisms mediated by the parahippocampal and associated regions (Yonelinas 2002). Since brain networks have easier access to the parahippocampal and associated regions than to the hippocampus due to the anatomical organization, respiratory modulation of familiarity or implicit memory likely occurs via networks outside hippocampus.

Our results showed that the right TPJa, right MFG, and dorsomedial prefrontal cortex had reduced activation during retrieval processes encompassing the EI transition, but the activation was higher during retrieval than during encoding or delay. Although the functional role of the activation pattern during retrieval remains unclear, the precise anatomical identification of the right TPJa (MNI coordinates: *x* = 58, *y* = −24, *z* = 38) may provide a clue regarding discrete functions in the brain. According to Mars et al. (2012), the right TPJa is well known as a neural core of the ventral attention network, which is a bottom-up attentional system involved in attentional control and in the awareness of salient events from the external environment (Corbetta et al. 2008; Asplund et al. 2010). The right MFG is the other core of the ventral attention network and may also be linked to the dorsal attention network (Corbetta et al. 2008). Igelström et al. (2015) indicated that the TPJa has preferential functional connectivity to the cingulo-opercular network (or salience network), whose neural hubs are the dACC and anterior insula/frontal operculum, suggesting that these regions are involved in tonic alertness and salience detection (Menon and Uddin 2010; van den Heuvel and Sporns 2013; Igelström and Graziano 2017). Accordingly, declines in task-related activity in the right TPJa, right MFG, and dorsomedial prefrontal cortex might represent the respiratory interference specific to information manipulation rather than memory storage.

The human TPJ has been suggested to be one of the most prominent sources of P300 components, which are event-related potentials associated with surprising events and contextual updating of representations (Nieuwenhuis et al. 2005; Corbetta et al. 2008; Mars et al. 2008). Interestingly, P300 components reflect the phasic activity of the locus coeruleus (Aston-Jones and Cohen 2005), which received projection fibers directly from the PreBötC (Yackle et al. 2017). It is tempting to speculate that respiratory modulation could be relevant to attention mechanisms based on the pathway from the PreBötC to the locus coeruleus to the TPJa to the MFG and dorsomedial prefrontal cortex during retrieval. Elucidation of detailed functional relationships of large-scale brain networks from the brainstem is a subject for future work using high-resolution neuroimaging. The present findings contribute to a better understanding of mind-brain interactions based on breathing for successful performance in daily life.

## Supplementary Material

FigsSup-NakamuraNH_tgac038

TableS1-NakamuraNH_tgac038

TableS2-NakamuraNH_tgac038

TableS3-NakamuraNH_tgac038

TableS4-NakamuraNH_tgac038

TableS5_6-NakamuraNH_tgac038

TableS7-NakamuraNH_tgac038
