# Supplementary material for: Respiration-timing-dependent changes in activation of neural substrates during cognitive processes
Source: Cereb Cortex Commun. 2022 Sep 13;3(4):tgac038. doi: 10.1093/texcom/tgac038 (PMC9552779; doi:10.1093/texcom/tgac038)
Supplement: FigsSup-NakamuraNH_tgac038 [file figssup-nakamuranh_tgac038.pdf]

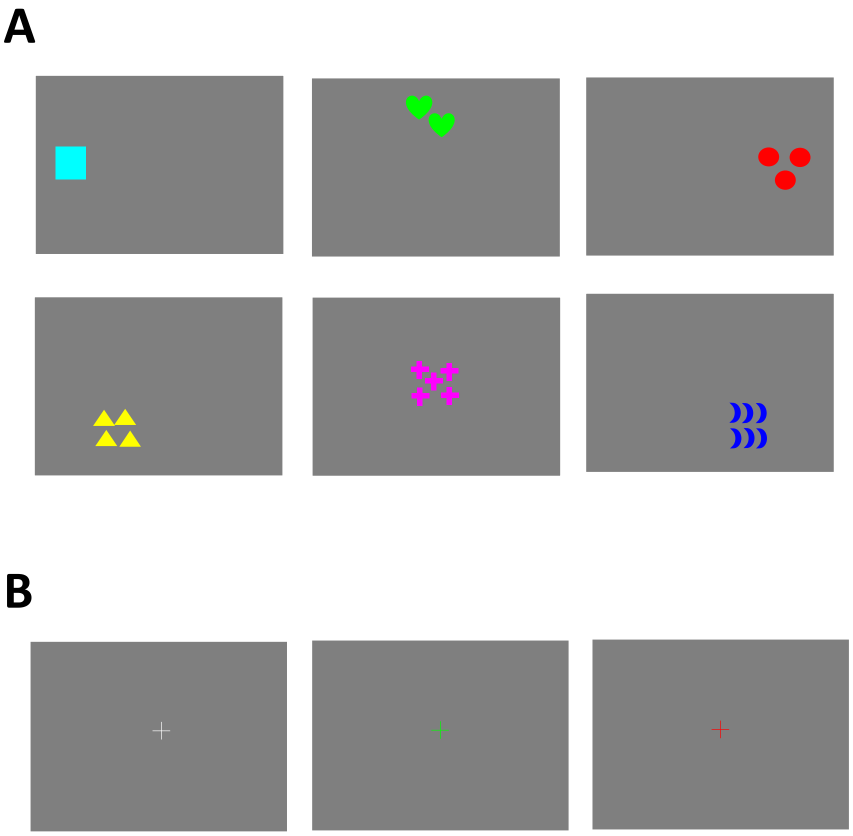

Supplementary Figure 1. Examples of visual cues during the DMTS task. A. Images showing representative visual cues on the screen during the sample and test sections: one sky-blue rectangle on the left (upper and left panel), two green hearts on the top center (upper and center panel), three red circles on the right (upper and right panel), four yellow triangles on the bottom right (lower and left panel), five pink crosses on the center (lower and center panel), and six blue crescents on the bottom left (lower and right panel). There were 1296 (6 x 6 x 6 x 6 variables) possible combinations. **B.** Subjects were instructed to fix their eyes to the white cross at the center of the screen. At the start of the sample section, the white cross (left panel) turned green (center panel) and then a series of four visual cues was exposed one at a time. At the start of the test section, the white cross turned red (right panel) and then a series of ten visual cues was exposed one at a time. Of note, a size of the cross was small enough to distinguish from visual cues.

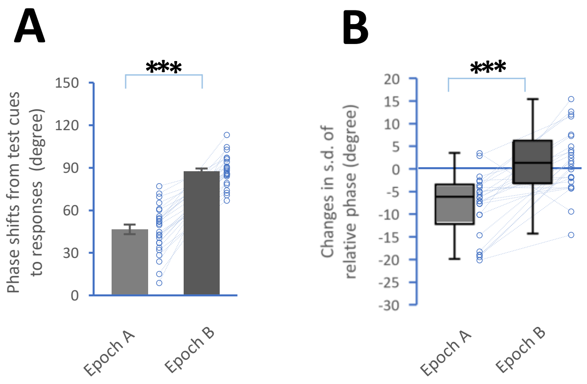

Supplementary Figure 2. Phase shifts and variability from test cues to motor responses in Epochs A and B.

A,B. Bar plots showing phase shifts (**A**) and changes in variability (i.e., standard deviation) (**B**) (mean ± s.e.m.; n = 25) from test cues to motor responses along the circular respiratory phases between Epochs A and B across individual participants. A finer gained analysis showed that phase shifts in Epoch A (46.5° , equivalent to 0.54 s of RT) were smaller than those in Epoch B (87.4° , equivalent to 1.02 s of RT) ($t(24) = 11.03$, $p < 0.00001$, two-tailed paired *t*-test, **A**). Changes in standard deviation of relative phase in Epoch A was lower than those in Epoch B ($p = 0.00001$, Wilcoxon signed rank test). The RT duration in Epoch A was more compressed in circular respiratory phases than on a real temporal scale (1.07 s in Epoch A and 1.10 s in Epoch B), indicating that the timing of the responses in Epoch A was biased to stay in the period ahead of the EI transition.

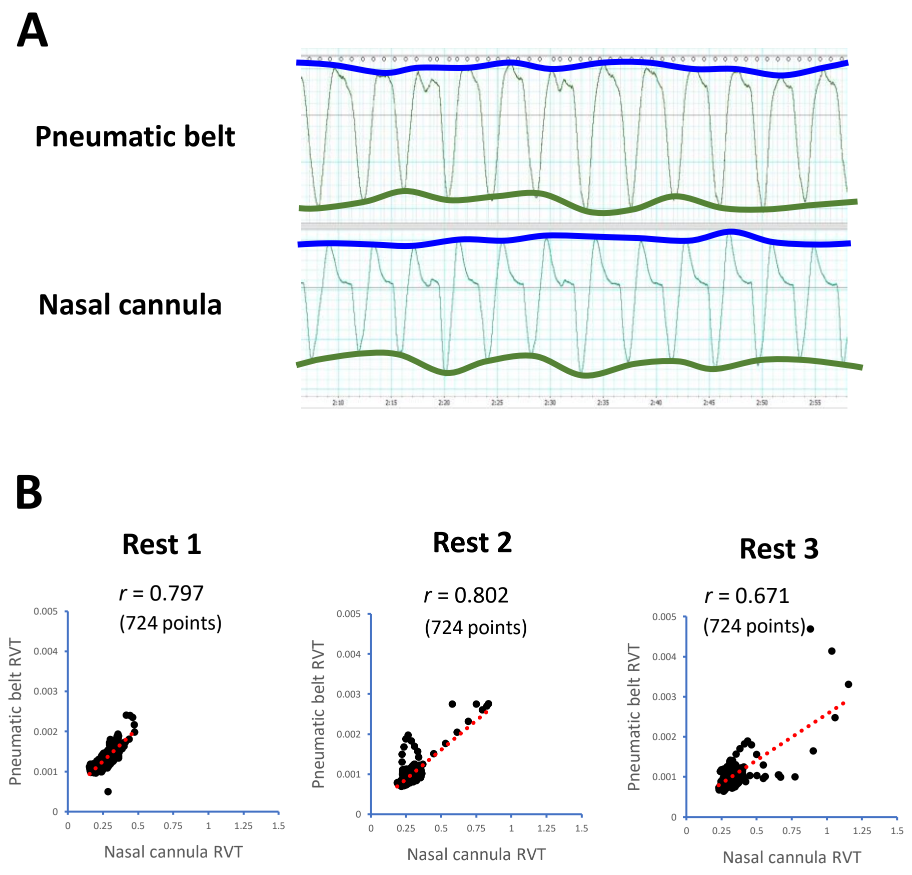

Supplementary Figure 3. The respiratory volume per time (RVT) parameters using pneumatic belt and nasal cannula. We simultaneously measured respiratory waveforms by pneumatic belt from the chest and nasal cannula with an extended polyvinyl chloride tube (in total of 7 m) for 10 min in resting states. **A.** Line graph showing a representative waveforms by pneumatic belt (on the top) and nasal cannula (on the bottom) with the envelope (blue lines) and bottom line (green lines) of respiration. The RVT parameters were calculated from the envelope and breath-to-breath variability of the respiratory depth and rate by AFNI's RetroTS program (see Methods). Then, they were averaged within each time window (0.8 s) with a 5.6-s delay. **B.** There were positive correlations between the RVT parameters obtained from the pneumatic belt and nasal cannula (r = 0.76 ± 0.04) in 10-min resting states. Then, the envelop from nasal cannula was applied as the RVT parameter for further analysis.

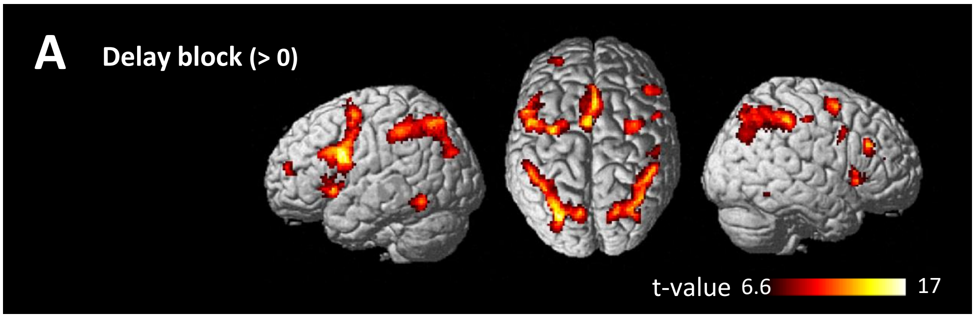

Supplementary Figure 4 fMRI activity during delay block in the DMTS task. A, Images showing brain regions that exhibited fMRI activity during the delay block (see Supplementary Table 3). fMRI activity is projected onto the cortical surface at a threshold for SPM{t} of p < 0.05 with family-wise error (FWE) correction at the peak level for the whole brain.
