# Supplementary material for: Respiration-timing-dependent changes in activation of neural substrates during cognitive processes
Source: Cereb Cortex Commun. 2022 Sep 13;3(4):tgac038. doi: 10.1093/texcom/tgac038 (PMC9552779; doi:10.1093/texcom/tgac038)
Supplement: TableS1-NakamuraNH_tgac038 [file tables1-nakamuranh_tgac038.docx]

| **Supplementary Table 1. The direction and dispersion of histograms of relative phase in test cues and button-press responses (mean ± s.d.)** | | | | |
| --- | --- | --- | --- | --- |
|  |  |  |  |  |
|  |  | Epoch A | Epoch B |  |
|  | Test cues | 223.8 ± 96.3° | 209.1 ± 71.7° |  |
|  | Button-press responses | 270.4 ± 88.4° | 296.5 ± 73.7° |  |
