# Supplementary material for: Respiration-timing-dependent changes in activation of neural substrates during cognitive processes
Source: Cereb Cortex Commun. 2022 Sep 13;3(4):tgac038. doi: 10.1093/texcom/tgac038 (PMC9552779; doi:10.1093/texcom/tgac038)
Supplement: TableS2-NakamuraNH_tgac038 [file tables2-nakamuranh_tgac038.docx]

**Supplementary Table 2. Brain regions that exhibited fMRI activity during the sample block.**

| Lobe | Peak level |  | MNI corrdinates (mm) | | |  | Region |
| --- | --- | --- | --- | --- | --- | --- | --- |
|  | t(24) | p(FEW-corr) | x | y | z | Side |  |
| **Sample block (> 0)** | | |  |  |  |  |  |
| Frontal | 7.52 | 0.007 | 42 | 30 | 12 | R | Inferior frontal gyrus (17%), MFG (12%) |
|  | 7.07 | 0.02 | 50 | 6 | 14 | R | Inferior frontal gyrus (17%), MI (15%) |
|  | 7.28 | 0.01 | 38 | 42 | 16 | R | MFG |
|  | 6.76 | 0.03 | -44 | 48 | 12 | L | MFG |
|  | 6.76 | 0.03 | -34 | 50 | 12 | L | MFG |
|  | 10.33 | <0.0001 | 26 | 0 | 60 | R | Superior frontal gyrus (48%), MFG (25%) |
|  | 13.41 | <0.0001 | -48 | 8 | 0 | L | Frontal operculum (18%), Central operculum (15%), Anterior insula (13%) |
|  | 9.81 | <0.0001 | 32 | 18 | 4 | R | Anterior insula (34%), Frontal operculum (19%) |
|  | 10.07 | <0.0001 | -4 | 4 | 28 | L | dACC |
|  | 6.69 | 0.03 | 6 | 0 | 30 | R | dACC |
|  | 14.15 | <0.0001 | -4 | 6 | 60 | L | preSMA |
|  |  |  |  |  |  |  |  |
| Temporal | 11.55 | <0.0001 | -50 | -54 | -10 | L | Inferior temporal gyrus |
|  | 8.66 | 0.001 | 54 | -56 | -12 | R | Inferior temporal gyrus |
|  | 7.15 | 0.01 | -56 | -54 | 14 | L | Middle temporal gyrus (36%), Superior temporal gyrus (33%) |
|  | 8.10 | 0.002 | -28 | -22 | -4 | L | Hippocampus (10%), Ventral Diencephalon (10%) |
|  |  |  |  |  |  |  |  |
| Parietal | 12.69 | <0.0001 | 44 | -38 | 46 | R | SMG (52%), Superior parietal lobule (18%) |
|  | 15.97 | <0.0001 | -30 | -62 | 54 | L | Superior parietal lobule (62%), Angular gyrus (22%) |
|  |  |  |  |  |  |  |  |
| Sub-lobar | 10.90 | <0.0001 | -16 | 2 | 16 | L | Caudate |
|  | 8.50 | 0.001 | 14 | 6 | 4 | R | Caudate (23%), Pallidum (13%) |
|  | 9.27 | <0.0001 | -14 | -36 | 0 | L | Thalamus |
|  | 7.24 | 0.01 | 8 | -4 | 0 | R | Thalamus |
|  | 6.89 | 0.02 | 12 | -10 | 6 | R | Thalamus |
|  | 6.87 | 0.02 | -12 | -14 | 6 | L | Thalamus |
|  |  |  |  |  |  |  |  |
| Cerebellum | 9.80 | <0.0001 | -12 | -70 | -22 | L | Cerebellum |
|  | 9.68 | <0.0001 | 42 | -62 | -28 | R | Cerebellum |
|  | 7.98 | 0.002 | -28 | -66 | -26 | L | Cerebellum |

dACC: Dorsal part of anterior cingulate cortex, MFG: Middle frontal gyrus, MI: Primary motor cortex, preSMA: Presupplementary motor area, SMG: Supramarginal gyrus, MNI: Montreal Neurological Institute (MNI) space, FWE-corr: family-wise error correction; The locations of local maxima are defined by the SPM Anatomical Toolbox. Reported results are *p* < 0.05 with family-wise error correction at the peak level for the whole brain.
