# Supplementary material for: Respiration-timing-dependent changes in activation of neural substrates during cognitive processes
Source: Cereb Cortex Commun. 2022 Sep 13;3(4):tgac038. doi: 10.1093/texcom/tgac038 (PMC9552779; doi:10.1093/texcom/tgac038)
Supplement: TableS3-NakamuraNH_tgac038 [file tables3-nakamuranh_tgac038.docx]

**Supplementary Table 3. Brain regions that exhibited fMRI activity during the delay block**

| Lobe | Peak level |  | MNI corrdinates (mm) | | |  | Region |
| --- | --- | --- | --- | --- | --- | --- | --- |
|  | t(24) | p(FEW-corr) | x | y | z | Side |  |
| **Delay block (> 0):** | | |  |  |  |  |  |
| Frontal | 7.49 | 0.007 | -28 | 52 | 18 | L | MFG (56%), Superior frontal gyrus (25%) |
|  | 9.38 | <0.0001 | 54 | 30 | 30 | R | MFG |
|  | 8.85 | <0.0001 | 36 | 0 | 56 | R | MFG (43%), MI (36%) |
|  | 6.70 | 0.03 | 40 | 42 | 16 | R | MFG |
|  | 12.32 | <0.0001 | -34 | 20 | 0 | L | Anterior insula (45%), Frontal operculum (28%) |
|  | 12.59 | <0.0001 | 38 | 20 | 4 | R | Frontal operculum (40%), Anterior insula (35%) |
|  | 13.93 | <0.0001 | 2 | 20 | 46 | LR | R-dACC (37%), L-dACC (28%) |
|  | 9.62 | <0.0001 | -4 | 6 | 28 | L | dACC |
|  | 7.92 | 0.003 | 6 | 2 | 30 | LR | R-dACC (26%), L-dACC (11%) |
|  | 6.75 | 0.03 | -14 | 26 | 32 | L | dACC |
|  | 8.13 | 0.002 | -4 | -22 | 26 | L | Midcingulate cortex |
|  | 7.10 | 0.02 | 6 | -24 | 28 | R | Midcingulate cortex |
|  | 8.14 | 0.002 | 52 | 10 | 38 | R | M1 (42%), MFG (30%) |
|  |  |  |  |  |  |  |  |
| Temporal | 12.89 | <0.0001 | -52 | -54 | -14 | L | Inferior temporal gyrus |
|  | 7.23 | 0.01 | 52 | -54 | -14 | R | Inferior temporal gyrus |
|  |  |  |  |  |  |  |  |
| Parietal | 7.67 | 0.005 | 52 | -20 | 48 | R | SI (51%), SMG (24%) |
|  | 12.65 | <0.0001 | 40 | -38 | 46 | R | SMG (38%), Superior parietal lobule (31%) |
|  | 16.93 | <0.0001 | -28 | -68 | 34 | L | Superior parietal lobule (17%), Angular gyrus (14%) |
|  | 7.81 | 0.003 | 24 | -66 | 24 | R | Precuneus (17%), Cuneus (15%) |
|  |  |  |  |  |  |  |  |
| Occipital | 7.40 | 0.009 | 12 | -72 | 16 | R | VI (31%), Cuneus (21%) |
|  |  |  |  |  |  |  |  |
| Sub-lobar | 10.50 | <0.0001 | -16 | 0 | 16 | L | Caudate |
|  | 7.65 | 0.005 | -18 | -30 | 8 | L | Thalamus |
|  | 7.14 | 0.01 | -10 | -22 | 16 | L | Thalamus |
|  | 6.95 | 0.02 | -8 | -6 | 6 | L | Thalamus |
|  | 8.09 | 0.002 | 14 | -16 | 10 | R | Thalamus |
|  | 7.81 | 0.003 | 12 | -14 | 0 | R | Thalamus |
|  | 6.73 | 0.03 | 8 | -6 | 18 | R | Thalamus (25%), Caudate (10%) |
|  |  |  |  |  |  |  |  |
| Cerebellum | 7.70 | 0.005 | -10 | -72 | -20 | L | Cerebellum |
|  | 7.04 | 0.02 | -22 | -46 | -26 | L | Cerebellum |
|  | 6.62 | 0.04 | -18 | -60 | -24 | L | Cerebellum |
|  | 6.67 | 0.04 | 0 | -52 | -10 | LR | Cerebellum |

dACC: Dorsal part of anterior cingulate cortex, MFG: Middle frontal gyrus, MI: Primary motor cortex, SMG: Supramarginal gyrus, SI: Primary somatosensory cortex, VI: Primary visual cortex, MNI: Montreal Neurological Institute (MNI) space, FWE-corr: family-wise error correction; The locations of local maxima are defined by the SPM Anatomical Toolbox. Reported results are *p* < 0.05 with family-wise error correction at the peak level for the whole brain.
