# Supplementary material for: Respiration-timing-dependent changes in activation of neural substrates during cognitive processes
Source: Cereb Cortex Commun. 2022 Sep 13;3(4):tgac038. doi: 10.1093/texcom/tgac038 (PMC9552779; doi:10.1093/texcom/tgac038)
Supplement: TableS4-NakamuraNH_tgac038 [file tables4-nakamuranh_tgac038.docx]

**Supplementary Table 4. Brain regions that exhibited fMRI activity during the test block**

| Lobe | Peak level |  | MNI corrdinates (mm) | | |  | Region |
| --- | --- | --- | --- | --- | --- | --- | --- |
|  | t(24) | p(FEW-corr) | x | y | z | Side |  |
| **Test block (> 0)** | | |  |  |  |  |  |
| Frontal | 6.74 | 0.04 | 30 | 44 | -6 | R | Anterior orbital gyrus |
|  | 7.12 | 0.02 | -40 | 42 | -2 | L | Inferior frontal gyrus (18%), MFG (17%) |
|  | 7.12 | 0.02 | -46 | 42 | 8 | L | Inferior frontal gyrus (37%), MFG (30%) |
|  | 6.80 | 0.03 | 46 | 44 | -8 | R | Inferior frontal gyrus (18%), Lateral orbital gyrus (15%) |
|  | 6.62 | 0.04 | 56 | 14 | 6 | R | Inferior frontal gyrus (43%), MI (10%) |
|  | 10.54 | <0.0001 | 28 | 52 | -2 | R | MFG |
|  | 7.99 | 0.002 | -26 | 54 | 14 | L | MFG (41%), Superior frontal gyrus (33%) |
|  | 6.78 | 0.03 | -38 | 44 | 18 | L | MFG |
|  | 6.62 | 0.04 | -34 | 38 | 20 | L | MFG |
|  | 7.19 | 0.01 | -36 | 26 | 12 | L | Frontal operculum |
|  | 8.40 | 0.001 | -10 | 30 | 28 | L | dACC |
|  | 7.81 | 0.003 | 8 | 38 | 16 | R | dACC |
|  | 13.70 | <0.0001 | -2 | 14 | 52 | LR | L-preSMA (51%), R-preSMA (21%) |
|  | 7.51 | 0.007 | 8 | -22 | 46 | R | Midcingulate cortex |
|  | 6.94 | 0.02 | 16 | -30 | 46 | R | Posterior cingulate cortex |
|  |  |  |  |  |  |  |  |
| Temporal | 6.82 | 0.03 | 52 | -54 | 6 | R | Middle temporal gyrus |
|  | 6.88 | 0.03 | 46 | -40 | 14 | R | Superior temporal gyrus |
|  | 6.74 | 0.04 | 54 | -36 | 12 | R | Superior temporal gyrus (35%), Middle temporal gyrus (12%) |
|  | 7.69 | 0.005 | 40 | -42 | -20 | R | Fusiform gyrus |
|  | 6.61 | 0.04 | 32 | -60 | -10 | R | Fusiform gyrus (16%), Occipital fusiform gyrus (16%) |
|  |  |  |  |  |  |  |  |
| Parietal | 7.77 | 0.004 | -62 | -18 | 18 | L | SI (27%), Central operculum (20%) |
|  | 7.14 | 0.02 | -18 | -42 | 44 | L | Precuneus |
|  |  |  |  |  |  |  |  |
| Occipital | 7.31 | 0.01 | 32 | -76 | -10 | R | Occipital fusiform gyrus |
|  | 7.55 | 0.006 | 40 | -66 | 12 | R | Middle occipital gyrus |
|  | 6.64 | 0.04 | 18 | -76 | -6 | R | Lingual gyrus |
|  |  |  |  |  |  |  |  |
| Sub-lobar | 8.08 | 0.002 | 14 | 10 | 4 | R | Caudate |
|  |  |  |  |  |  |  |  |
| Cerebellum | 16.87 | <0.0001 | -26 | -48 | -28 | L | Cerebellum |
|  | 7.95 | 0.003 | 42 | -24 | -28 | R | Cerebellum |

dACC: Dorsal part of anterior cingulate cortex, MFG: middle frontal gyrus, MI: Primary motor cortex, preSMA: Presupplementary motor area, SI: Primary somatosensory cortex. MNI: Montreal Neurological Institute (MNI) space, FWE-corr: family-wise error correction; The locations of local maxima are defined by the SPM Anatomical Toolbox. Reported results are *p* < 0.05 with family-wise error correction at the peak level for the whole brain.
