# Supplementary material for: Respiration-timing-dependent changes in activation of neural substrates during cognitive processes
Source: Cereb Cortex Commun. 2022 Sep 13;3(4):tgac038. doi: 10.1093/texcom/tgac038 (PMC9552779; doi:10.1093/texcom/tgac038)
Supplement: TableS5_6-NakamuraNH_tgac038 [file tables5_6-nakamuranh_tgac038.docx]

**Supplementary Table 5. Brain regions that exhibited fMRI activity contrasting Sample block with Test block**

| Lobe | Cluster level | |  | Peak level |  |  | MNI corrdinates (mm) | | |  | Region |
| --- | --- | --- | --- | --- | --- | --- | --- | --- | --- | --- | --- |
|  | Cluster size | p(FEW-corr) |  | t(24) | p(FEW-corr) |  | x | y | z | Side |  |
| **Sample block > Test block** | | |  |  |  |  |  |  |  |  |  |
| Parietal | 322 | 0.001 |  | 5.81 | 0.198 |  | -46 | -26 | 62 | L | SI |
|  |  |  |  | 5.51 | 0.33 |  | -32 | -28 | 54 | L | SI |

SI: Primary somatosensory cortex. MNI: Montreal Neurological Institute (MNI) space, FWE-corr: family-wise error correction; The locations of local maxima are defined by the SPM Anatomical Toolbox. Reported results are *p* < 0.05 with family-wise error correction at the cluster level for the whole brain.

**Supplementary Table 6. Brain regions that exhibited fMRI activity contrasting Test block with Sample block**

| Lobe | Peak level |  | MNI corrdinates (mm) | | |  | Region |
| --- | --- | --- | --- | --- | --- | --- | --- |
|  | t(24) | p(FEW-corr) | x | y | z | Side |  |
| **Test block > Sample block:** | | |  |  |  |  |  |
| Frontal | 7.1 | 0.018 | 44 | 44 | -8 | R | Lateral orbital gyrus (20%), Inferior frontal gyrus (15%) |
|  | 6.88 | 0.027 | -40 | 42 | -2 | L | Inferior frontal gyrus (18%), MFG (17%) |
|  | 5.56 | 0.049 | -34 | 18 | 22 | L | MFG |
|  | 6.58 | 0.047 | -32 | 22 | 22 | L | MFG |
|  | 6.66 | 0.041 | -34 | 50 | 24 | L | MFG |
|  | 7.15 | 0.016 | -30 | 12 | 48 | L | MFG |
|  | 10.28 | 0.0001 | -26 | 2 | 58 | L | MFG (46%), Superior frontal gyrus (38%) |
|  | 6.95 | 0.023 | -26 | 56 | -2 | L | MFG (29%), Superior frontal gyrus (17%) |
|  | 7.43 | 0.008 | -26 | 44 | 10 | L | MFG (41%), Superior frontal gyrus (30%) |
|  | 6.91 | 0.025 | 42 | 54 | 10 | R | MFG |
|  | 7.38 | 0.009 | 20 | 46 | 12 | R | MFG |
|  | 11.88 | 0.0001 | 28 | 52 | -2 | R | MFG |
|  | 6.92 | 0.025 | -12 | 4 | 62 | L | Superior frontal gyrus |
|  | 7.62 | 0.005 | -8 | 0 | 70 | LR | Superior frontal gyrus |
|  | 6.73 | 0.035 | 18 | 50 | 20 | R | Superior frontal gyrus |
|  | 7.19 | 0.014 | 12 | -6 | 72 | R | Superior frontal gyrus |
|  | 8.36 | 0.001 | -58 | -24 | 18 | L | Parietal operculum (21%), Central operculum(18%) |
|  | 7.08 | 0.018 | 58 | 0 | 6 | R | Central operculum |
|  | 8.3 | 0.001 | -10 | 30 | 28 | L | dACC |
|  | 6.98 | 0.023 | -8 | 38 | 20 | LR | dACC |
|  | 8.15 | 0.002 | 6 | 40 | 16 | LR | dACC |
|  | 11.12 | 0.0001 | 6 | -2 | 54 | LR | SMA |
|  | 8.07 | 0.002 | 8 | -22 | 46 | LR | Midcingulate cortex |
|  | 13.26 | 0.0001 | 6 | -28 | 28 | LR | Posterior cingulate cortex (14%), Midcingulate cortex (12%) |
|  | 6.65 | 0.041 | 14 | -34 | 40 | R | Posterior cingulate cortex |
|  | 6.67 | 0.04 | 16 | -32 | 46 | R | Posterior cingulate cortex |
|  |  |  |  |  |  |  |  |
| Temporal | 6.74 | 0.035 | 58 | -62 | -4 | R | Middle temporal gyrus (29%), Inferior temporal gyrus (26%) |
|  | 6.64 | 0.042 | -50 | -22 | -4 | L | Superior temporal gyrus (22%), Middle temporal gyrus (16%) |
|  | 7 | 0.021 | 52 | -16 | -8 | R | Superior temporal gyrus (34%), Middle temporal gyrus (24%) |
|  | 6.84 | 0.029 | 28 | -4 | -14 | R | Amygdala (12%), Putamen (9%) |
|  | 6.91 | 0.025 | 32 | -60 | -10 | R | Fusiform gyrus |
|  | 7.65 | 0.005 | 40 | -42 | -20 | R | Fusiform gyrus |
|  |  |  |  |  |  |  |  |
| Parietal | 6.9 | 0.026 | -38 | -26 | 34 | L | SI |
|  | 14.51 | 0.0001 | 52 | -22 | 50 | R | SI (43%), SMG (29%) |
|  | 6.59 | 0.046 | 28 | -38 | 64 | R | Superior parietal lobule |
|  | 6.86 | 0.028 | -12 | -66 | 56 | L | Superior parietal lobule |
|  | 6.87 | 0.027 | 10 | -50 | 48 | R | Precuneus |
|  | 6.89 | 0.026 | -6 | -60 | 56 | LR | Precuneus |
|  | 8.06 | 0.002 | 0 | -56 | 48 | LR | Precuneus |
|  | 8.66 | 0.001 | -16 | -42 | 44 | L | Precuneus |
|  |  |  |  |  |  |  |  |
| Occipital | 7.55 | 0.006 | 40 | -66 | 12 | R | Middle occipital gyrus (12%), Inferior occipital gyrus (8%) |
|  | 6.93 | 0.024 | 30 | -76 | -10 | R | Occipital fusiform gyrus |
|  |  |  |  |  |  |  |  |
| Sub-lobar | 6.79 | 0.032 | -14 | 8 | 4 | L | Caudate |
|  | 10.11 | 0.0001 | -28 | 12 | 4 | L | Putamen (29%), Anterior insula (23%) |
|  | 12.51 | 0.0001 | 32 | -8 | -2 | R | Putamen |
|  | 6.87 | 0.027 | 0 | 0 | 10 | LR | Thalamus |
|  | 7.65 | 0.005 | 2 | -14 | -12 | LR | Ventral Diencephalon |
|  |  |  |  |  |  |  |  |
| Cerebellum | 16.63 | 0.0001 | -26 | -48 | -28 | L | Cerebellum |
|  | 6.58 | 0.047 | 6 | -62 | -6 | R | Cerebellum |

dACC: Dorsal part of anterior cingulate cortex, MFG: middle frontal gyrus, MI: Primary motor cortex, SMA: Supplementary motor area, SMG: Supramarginal gyrus, SI: Primary somatosensory cortex. MNI: Montreal Neurological Institute (MNI) space, FWE-corr: family-wise error correction; The locations of local maxima are defined by the SPM Anatomical Toolbox. Reported results are *p* < 0.05 with family-wise error correction at the peak level for the whole brain.
