# Supplementary material for: Respiration-timing-dependent changes in activation of neural substrates during cognitive processes
Source: Cereb Cortex Commun. 2022 Sep 13;3(4):tgac038. doi: 10.1093/texcom/tgac038 (PMC9552779; doi:10.1093/texcom/tgac038)
Supplement: TableS7-NakamuraNH_tgac038 [file tables7-nakamuranh_tgac038.docx]

**Supplementary Table 7. Brain regions that exhibited fMRI activity contrasting between the conditions.**

| Lobe | Cluster level | |  | Peak level |  |  | MNI corrdinates (mm) | | |  | Region |
| --- | --- | --- | --- | --- | --- | --- | --- | --- | --- | --- | --- |
|  | Cluster size | p(FEW-corr) |  | t(24) | p(FEW-corr) |  | x | y | z | Side |  |
| **IE > EI** | | |  |  |  |  |  |  |  |  |  |
| Parietal | 141 | 0.003 |  | 4.63 | 0.938 |  | 58 | -24 | 38 | R | TPJa |
|  |  |  |  | 4.51 | 0.969 |  | 62 | -18 | 44 | R | TPJa |
|  |  |  |  |  |  |  |  |  |  |  |  |
| **INS + EXP > EI** | | |  |  |  |  |  |  |  |  |  |
| Frontal | 148 | 0.003 |  | 5.3 | 0.523 |  | -34 | 50 | 26 | L | MFG |
|  |  |  |  | 4.85 | 0.83 |  | -28 | 52 | 28 | L | MFG |
|  |  |  |  | 4.15 | 0.999 |  | -18 | 36 | 32 | L | Superior frontal gyrus (21%), MFG (11%) |
|  | 116 | 0.01 |  | 5.04 | 0.708 |  | 38 | 36 | 22 | R | MFG |
|  |  |  |  | 4.76 | 0.889 |  | 46 | 48 | 16 | R | MFG |
|  |  |  |  | 3.63 | 1 |  | 44 | 34 | 12 | R | Inferior frontal gyrus (28%), MFG (26%) |
|  | 2245 | <0.0001 |  | 6.43 | 0.076 |  | -60 | -16 | 28 | L | SI |
|  |  |  |  | 6.16 | 0.125 |  | -40 | -4 | 16 | L | Central operculum (29%), Anterior insula (12%) |
|  |  |  |  | 6.01 | 0.164 |  | -42 | -4 | 10 | L | Central operculum (41%), Anterior insula (21%) |
|  | 1056 | <0.0001 |  | 6.20 | 0.116 |  | -8 | 12 | 42 | L | dACC |
|  |  |  |  | 5.79 | 0.244 |  | -2 | -6 | 62 | LR | L-SMA (45%), R-SMA (22%) |
|  |  |  |  | 5.59 | 0.339 |  | -2 | 16 | 32 | LR | L-dACC (43%), R-dACC (25%) |
|  | 173 | 0.001 |  | 6.16 | 0.124 |  | -34 | -8 | 58 | L | MI |
|  |  |  |  | 4.23 | 0.997 |  | -44 | -10 | 54 | L | MI |
|  |  |  |  |  |  |  |  |  |  |  |  |
| Parietal | 4888 | <0.0001 |  | 7.38 | 0.009 |  | 56 | -16 | 48 | R | SI (48%), SMG (12%) |
|  |  |  |  | 6.86 | 0.031 |  | 38 | -38 | 38 | R | SMG (20%), Superior parietal lobule (13%) |
|  |  |  |  | 6.58 | 0.057 |  | 46 | -22 | 50 | R | SI (54%), SMG (15%) |
|  |  |  |  |  |  |  |  |  |  |  |  |
| Temporal | 6599 | <0.0001 |  | 7.5 | 0.007 |  | -40 | -64 | -10 | L | Inferior temporal gyrus (17%), Inferior occipital gyrus (13%) |
|  |  |  |  | 7.33 | 0.01 |  | 46 | -58 | -4 | R | Inferior temporal gyrus (24%) |
|  |  |  |  | 7.32 | 0.011 |  | -38 | -60 | -10 | L | Fusiform gyrus (19%), Inferior temporal gyrus (13%) |
|  |  |  |  |  |  |  |  |  |  |  |  |
| Occipital | 100 | 0.021 |  | 4.97 | 0.758 |  | 44 | -82 | -4 | R | Inferior occipital gyrus |
|  |  |  |  | 4.87 | 0.819 |  | 40 | -78 | -8 | R | Inferior occipital gyrus |
|  |  |  |  | 3.94 | 1 |  | 36 | -84 | 6 | R | Inferior occipital gyrus (40%), Middl occipital gyrus (14%) |

dACC: Dorsal part of anterior cingulate cortex, MFG: Middle frontal gyrus, MI: Primary motor cortex, SMA: Supplementary motor area, SMG: Supramarginal gyrus, SI: Primary somatosensory cortex, TPJa: Anterior part of temporoparietal junction, MNI: Montreal Neurological Institute (MNI) space, FWE-corr: family-wise error correction; The locations of local maxima are defined by the SPM Anatomical Toolbox. Reported results are *p* < 0.05 with family-wise error correction at the cluster level for the whole brain.
